# T75. GENERAL AND EXECUTIVE COGNITIVE PROFILES: GENERAL COGNITIONS INFLUENCE ON WCST PERFORMANCE

**DOI:** 10.1093/schbul/sby016.351

**Published:** 2018-04-01

**Authors:** Sean Carruthers, Caroline Gurvich, Philip Sumner, Eric Tan, Elizabeth Thomas, Susan Rossell

**Affiliations:** 1 Swinburne University; 2 The Alfred Hospital, Monash University; 3 Swinburne University, The Alfred Hospital; 4 The Alfred Hospital, Monash University, Swinburne University

## Abstract

**Background:**

Executive functions (EF) have been conceptualized as a set of higher-level control processes that enable an individual to adapt to diverse situations, inhibit inappropriate responses, formulate, initiate and persevere plans and mediate the organisation of goal-directed thoughts and actions and are commonly reported as being compromised in schizophrenia. Complex measures designed to assess EF suffer from task impurity, activating and reporting on the performance of non-EF processes. The present study examined the potential contribution discrete cognitive subtypes might have on performance on the Wisconsin Card Sorting Test

**Methods:**

Ward’s method hierarchical cluster analysis was performed on the MATRICS Consensus Cognitive Battery (MCCB) and the Wisconsin Card Sorting Test (WCST) collected from 105 healthy controls and 100 patients with schizophrenia/schizoaffective disorder.

**Results:**

Two cognitive profiles were identified for general cognition (High/Low) and the WCST (High/Low). For controls, only 53% of low performing participants performed low on the WCST. For patients, 73.5% of patients who performed poorly on the MCCB were found to perform poorly on the WCST.

**Discussion:**

Results indicate that the contribution of general cognitive domains on poor WCST performance differs between patients and control participants. For patients, there appears to be a substantial contribution of impaired general cognition to performance the WCST.

